# Establishment of a repeated social defeat stress model in female mice

**DOI:** 10.1038/s41598-017-12811-8

**Published:** 2017-10-09

**Authors:** Aki Takahashi, Jia-Ru Chung, Song Zhang, Hongxing Zhang, Yael Grossman, Hossein Aleyasin, Meghan E. Flanigan, Madeline L. Pfau, Caroline Menard, Dani Dumitriu, Georgia E. Hodes, Bruce S. McEwen, Eric J. Nestler, Ming-Hu Han, Scott J. Russo

**Affiliations:** 1 Fishberg Department of Neuroscience and Friedman Brain Institute, Icahn School of Medicine at Mount Sinai, New York, New York, 10029 United States; 2 Laboratory of Behavioral Neuroendocrinology, University of Tsukuba, Tsukuba, Ibaraki 305-8577 Japan; 3 Laboratory of Neuroendocrinology, The Rockefeller University, New York, NY 10065 United States; 4 Department of Pharmacological Sciences and Institute for Systems Biomedicine, Icahn School of Medicine at Mount Sinai, New York, New York, 10029 United States

## Abstract

Numerous studies have employed repeated social defeat stress (RSDS) to study the neurobiological mechanisms of depression in rodents. An important limitation of RSDS studies to date is that they have been conducted exclusively in male mice due to the difficulty of initiating attack behavior directed toward female mice. Here, we establish a female mouse model of RSDS by inducing male aggression toward females through chemogenetic activation of the ventrolateral subdivision of the ventromedial hypothalamus (VMHvl). We demonstrate that females susceptible to RSDS display social avoidance, anxiety-like behavior, reduction of body weight, and elevated levels of circulating interleukin 6. In contrast, a subset of mice we term resilient only display anxiety-like behaviors after RSDS. This model allows for investigation of sex differences in the neurobiological mechanisms of defeat‒induced depression‒like behaviors. A robust female social defeat model is a critical first step in the identification and development of novel therapeutic compounds to treat depression and anxiety disorders in women.

## Introduction

Violence against women is a major public health problem—an estimated 35% of women worldwide have experienced physical and/or sexual violence at some point in their lives^1^. Women who are victims of violence are more likely to develop mental illnesses including mood disorders and attempt suicide at higher rates^2,3^. Mood disorders are one of the most common mental health disorders, and women are more than twice as likely as men to develop depression and anxiety disorders^4–7^. In addition to being at increased risk for mood disorders, women exhibit different symptomatic profiles and comorbidities than men. While women are more likely to experience comorbid anxiety, men show higher comorbidity with substance abuse^8^. There is also increasing evidence of sex differences in the underlying biology of stress-related disorders in men and women^9–11^. Despite these important issues, the majority of preclinical studies of stress-related disorders focus heavily on male rodents.

The RSDS model has been employed extensively in male rodents to study neurobiological mechanisms of depression, as the model has strong ethological significance and face validity to human mood disorders, distinguishing it from other preclinical models of depression^12–18^. It is well established in humans that repeated experience of social stress, such as bullying or physical abuse, increases risk of developing depression^19^. Much like humans, socially defeated intruder male mice develop several depressive-like phenotypes characterized by social avoidance, reduction of sexual motivation, anhedonia, behavioral despair, reduction of body weight, risk for atherosclerosis, changes in hormonal and cardiovascular responses and enhanced pro-inflammatory responses^12,17,18,20–25^. RSDS also has pharmacological validity as chronic, but not acute, treatment of mice with standard antidepressants normalizes the behavioral effects of defeat^17,26^. Of further utility, RSDS yields a pool of rodents that exhibit large individual differences in response to defeat, with 50-60% of mice displaying stress susceptibility and about 30% of mice displaying stress resilience^17,18^, allowing for the study of biological mechanisms underlying susceptibility and resilience to social stress^27,28^. Identification of active biological coping mechanisms in resilient mice has spurred investigation of novel therapeutics to promote natural resilience mechanisms as an alternative to traditional anti-depressant drug development^29,30^.

Nevertheless, an important caveat of the RSDS model is its reliance on a male’s innate territorial aggression toward other males that intrude into his territory. Because aggression toward sexually matured female mice (*Mus musculus*) were rare or low compared to inter-male aggression, RSDS studies to date have been conducted exclusively in male mice. In the present study, we aimed to establish a female RSDS model by inducing male aggression toward adult female mice through a chemogenetic approach. The hypothalamic attack area is a locus tightly linked to attack behavior^31,32^. Previous studies have shown that activation of the ventrolateral subdivision of the ventromedial hypothalamus (VMHvl) in male mice induces atypical attack behavior in which male mice attacked female intruders as well as inanimate objects^33,34^. Here, we used designer receptors exclusively activated by designer drugs (DREADDs) to activate the VMHvl of male mice, promoting aggression toward adult females and enabling successful RSDS outcomes in female mice. Our results describe a reliable and reproducible method of RSDS to induce depression-like behavioral responses in both female and male mice.

## Results

### Establishment of aggressive males that attack female mice

**Figure 1:**
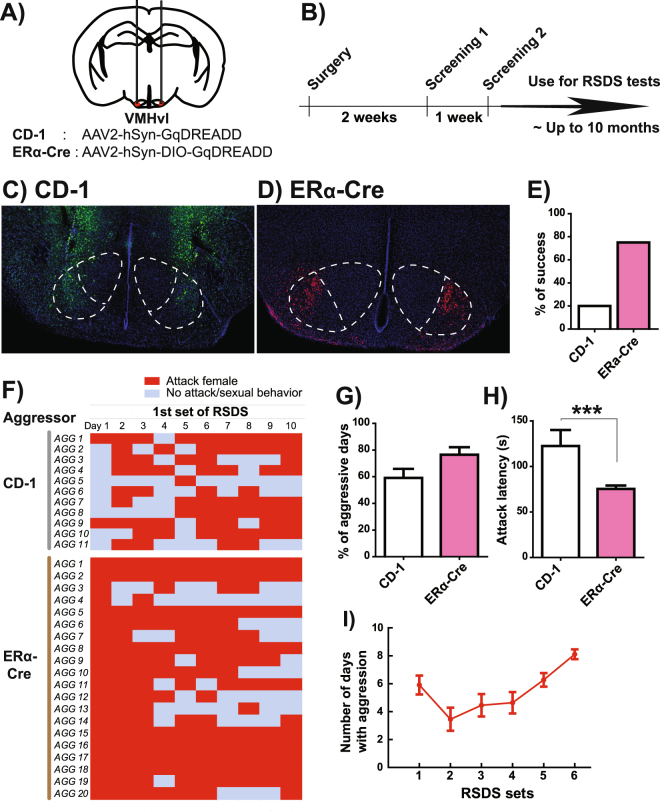
Generation of male aggressors to defeat female mice. (**A**) Schematics of Gq-DREADD expressing AAV injection bilaterally into VMHvl. AAV2-hSyn-GqDREADD-IRES-mCitrine and AAV2-hSyn-DIO-GqDREADD-mCherry were injected into male wild-type CD-1 and ERα-Cre mice, respectively. (**B**) Time course of surgery and screening of aggressive behavior. (**C**) Representative picture of CD-1 male with Gq-DREADD injections into the VMHvl. mCitrine expression (green) is not restricted to the VMHvl but is widely spread around the VMH area. (**D**) Representative picture of ERα-Cre F1 male with Gq-DREADD injections into the VMHvl. mCherry expression (red) is localized in the VMHvl area. (**E**) Percentage of males that showed aggressive behavior toward female C57BL/6J intruders during the aggression screening. (**F**) Daily monitoring of aggressive behavior during the first 10-day RSDS procedure in each aggressor male. (**G**) Number of days that males showed aggressive behaviors and (**H**) average attack latency of aggressor males during 10-day RSDS. (**I**) The same CD-1 aggressor males were used for several iterations of RSDS without reducing aggressive behavior. Data represent mean ± SEM, ***p < 0.001 in unpaired t-test.

To establish male aggression toward adult, sexually mature female mice, we first injected Gq-DREADD expressing AAV bilaterally into the VMHvl of CD-1 male mice (Fig. 1A,B). We screened these animals for aggression toward female mice both two and three weeks post-surgery. For screening, CD-1 males were given intraperitoneal injections of 1.0 mg/kg clozapine-N-oxide (CNO). Thirty-five minutes later, a C57BL/6J female was introduced into the homecage of a CD-1 male to screen his aggressive behavior. Under these conditions, approximately 20% of CD-1 males (21 out of 105 males) showed consistent aggressive behavior toward females during the screening and were used for the RSDS procedure (Fig. 1E). Histological examination indicated that animals lacking attack behavior had low or no infection of virus in the VMHvl area as only 20% of mice showing aggression had expression of AAV-Gq-DREADD within the VMHvl (Fig. 1C).

To increase the efficiency of targeting the VMHvl, we used estrogen receptor alpha (ERα)-Cre mice in which Cre expression is highly localized to the VMHvl and absent in adjacent structures^34^. Previous work has implicated this ERα-positive neuronal population in aggressive behaviors^34,35^. To ensure consistent aggression, we crossed ERα-Cre mice with CD-1 outbred mice used in standard male RSDS protocols to obtain F1 offspring with the ERα-Cre allele^36^. We then injected Cre-dependent DIO-Gq-DREADD-expressing AAV into bilateral VMHvl in F1 ERα-Cre males and subsequently screened aggression toward a female intruder mouse 2 and 3 weeks after viral injection (Fig. 1A,B). Remarkably, 75% of these mice (30 out of 40 males) exhibited CNO-induced aggressive behavior toward females during the screening test (Fig. 1D,E).

Quantitatively, we observed greater aggression toward females in Gq-DREADD-injected ERα-Cre mice compared to Gq-DREADD-injected CD-1 mice. CD-1 male mice exhibited aggressive behavior toward females for an average of 59.1% of each defeat session (ranging from 10 to 90%) whereas ERα-Cre mice exhibited aggressive behavior for an average of 76.5% of each defeat session (Fig. 1F,G). Latency to attack initiation was shorter in ERα-Cre compared to CD-1 aggressors (Fig. 1H). Aggressive behavioral patterns in both CD-1 and ERα-Cre aggressor males were similar to inter-male aggressive behavior (Supplemental Videos 1 and 2).

Gq-DREADD-injected aggressors can be used repeatedly in the RSDS paradigm for up to 10 months and for as many as 6 separate RSDS studies with consistent levels of CNO-induced aggression (Fig. 1I, CD-1 aggressors). Thus, chronic Gq-DREADD activation of VMHvl neurons produces reliable, stable aggression toward intruder female mice.

### Characterization of a conventional RSDS model in females

**Figure 2:**
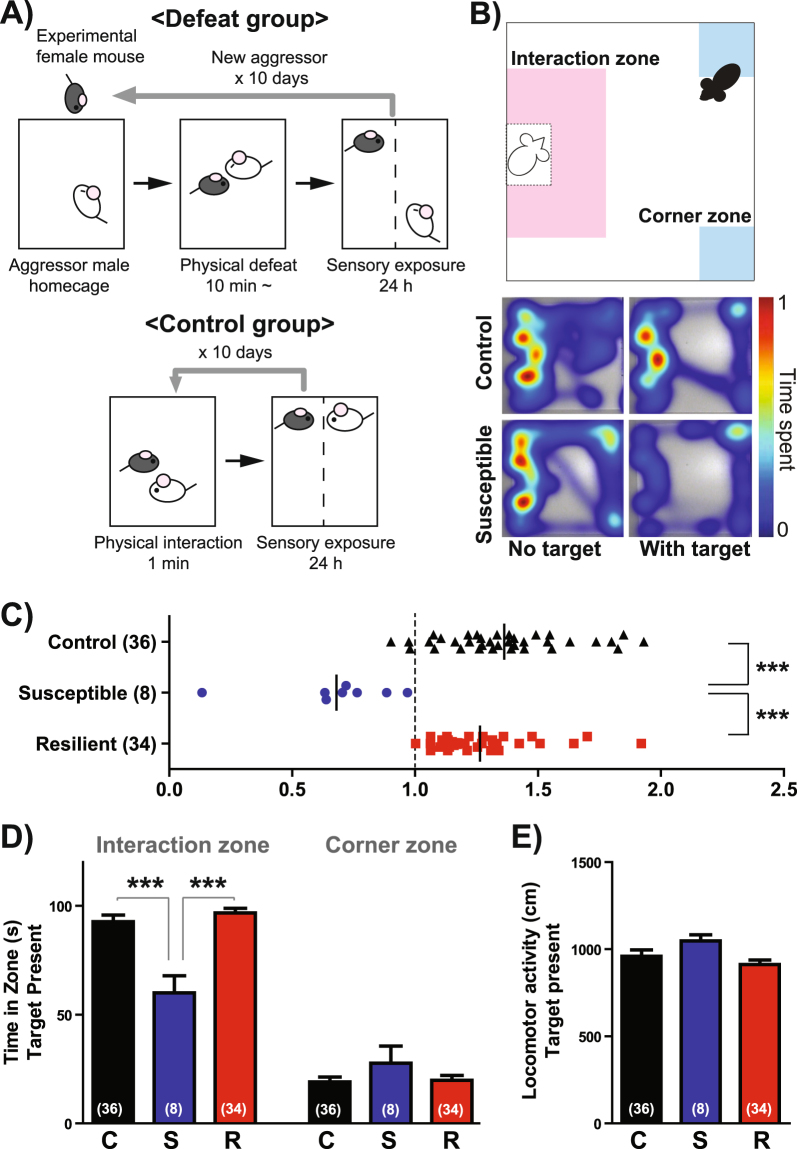
Conventional RSDS produces social avoidance in a small fraction of female mice. (**A**) Experimental schematics of conventional RSDS applied to female C57BL/6J mice. (**B**) Social interaction (SI) testing apparatus depicting the interaction zone (pink) and corner zone (blue) (**top**). Representative traces of the exploratory behavior of control and susceptible female mice in the presence and absence of a novel male mouse (**bottom**). (**C**) Distribution of SI ratio in control and defeated females. Defeated females that showed social avoidance (SI ratio <1) were categorized as susceptible, and females that showed social preference (SI ratio ≥1) were categorized as resilient. (**D**) Time spent in the interaction zone and corner zone and (**E**) locomotor activity when the social target was present in the arena. C: control, S: susceptible, R: resilient. Data represent mean ± SEM. Number of animals is indicated in parentheses. *p < 0.05; **p < 0.01; ***p < 0.001 in Tukey’s *post hoc* test after One-way ANOVA.

We first tested a conventional RSDS protocol based upon our previous studies in male C57BL/6J mice^12^. Female C57BL/6J mice were introduced into the homecage of a CNO-injected aggressor for ~10 min following an initial attack. Under these circumstances, we observed inconsistent CNO-induced attack behavior, finding that females were defeated on about 5.9 days (ranging from 2 to 10 days) or 9.6 days (ranging from 9 to 10 days) of the 10-day RSDS protocol in CD-1 or ERα-Cre aggressors, respectively. After each 10-minute defeat bout, the female mouse was placed on the opposite side of a Plexiglas divider for 24 hours until the next physical defeat bout, allowing for persistent sensory contact with the male aggressor as a form of psychological stress (Fig. 2A). Twenty-four hours after the last defeat bout, we assayed female social interaction behavior by performing a social interaction (SI) test as previously described^12^. During this 5-minute SI test, we record an animal’s exploratory behavior in an open-field arena containing a small wire enclosure that is empty (first 2.5 minutes) or houses a novel CD-1 male (second 2.5 minutes) (Fig. 2B). The ratio of time spent in the immediate vicinity of the wire enclosure (i.e., the interaction zone) when the target mouse is present over the time spent near the enclosure when the target mouse is absent serves as an index of social avoidance/approach and is termed SI ratio. Similar to the findings of studies employing male RSDS^17,18^, we found a large distribution of SI ratio in defeated females compared to controls (Fig. 2C). The distribution of SI ratios in defeated females was not normal (KS normality test: p = 0.0106), and thus we split the defeated population into stress-resilient (SI ratio > 1.0) and stress susceptible groups (SI ratio < 1.0). Interestingly, less than 20% of defeated females showed social avoidance (susceptible) in this test using either CD-1 (19%, Fig. 2C) or ERα-Cre F1 aggressors (10%, Supplemental Fig. 1). This percentage is quite low compared to inter-male RSDS, in which approximately 60% of males become susceptible^18^. Despite this low level of susceptibility, one-way ANOVA indicated a significant group differences for SI ratio (Fig. 2C; F(2,77) = 28.31, p < 0.0001) and time spent in interaction zone (Fig. 2D; F(2,66) = 16.32, p < 0.0001), whereby susceptible females exhibited lower SI ratios and time spent in interaction zone compared to both control and resilient females. In contrast, there were no differences between groups in time spent in the corner zone or in locomotor activity. The number of defeated days was not different between resilient and susceptible females (Supplemental Fig. 2a), and there was no correlation between SI ratio and the number of defeated days (Supplemental Fig. 2b) or the number of attack bouts received during 10-day RSDS (Supplemental Fig. 2c). Also, we did not observe any effect of estrous cycle stage on SI ratio in control or defeated female mice (Supplemental Fig. 2d,e).

A possible explanation for the surprisingly high rate of resilience in females exposed to conventional RSDS is the confound of sexual motivation. When we initiated female RSDS according to the conventional model, we noted that females, unlike males because of their larger body size, were able to climb over the Plexiglas divider and enter the opposite side of the defeat cage. Interestingly, many of the defeated females moved to the male’s compartment after the pro-aggression effect of CNO had subsided, suggesting that, unlike male C57BL/6J mice exposed to RSDS, defeated females do not find the 24-hour psychosensory exposure period aversive. It is well known that sensory exposure to male pheromones alters the behavior and sexual receptivity of female mice. Male mice also emit ultrasonic vocalizations that can attract females^37^. To examine the effect of sensory contact itself on RSDS behavioral outcome, we prepared two types of control groups: a female mouse co-housed with another female or co-housed with a male for 10 days (Supplemental Fig. 3a). Similar to the RSDS procedure, we co-housed these animals using a perforated divider to provide sensory, but not physical, contact. We found that, compared to females co-housed with females, control females co-housed with males exhibited higher body weight gain and water intake (Supplemental Fig. 3b,c), as well as reduced total arm entry in the elevated plus maze (Supplemental Fig. 3f) and reduced immobility in forced swim test (Supplemental Fig. 3i). There was no effect of co-housing on social interaction behavior in SI test, % of open arm entry in the elevated plus maze, and sucrose preference (Supplemental Fig. 3d,e,g,h).

### Characterization of an adapted RSDS model without extended sensory contact

**Figure 3:**
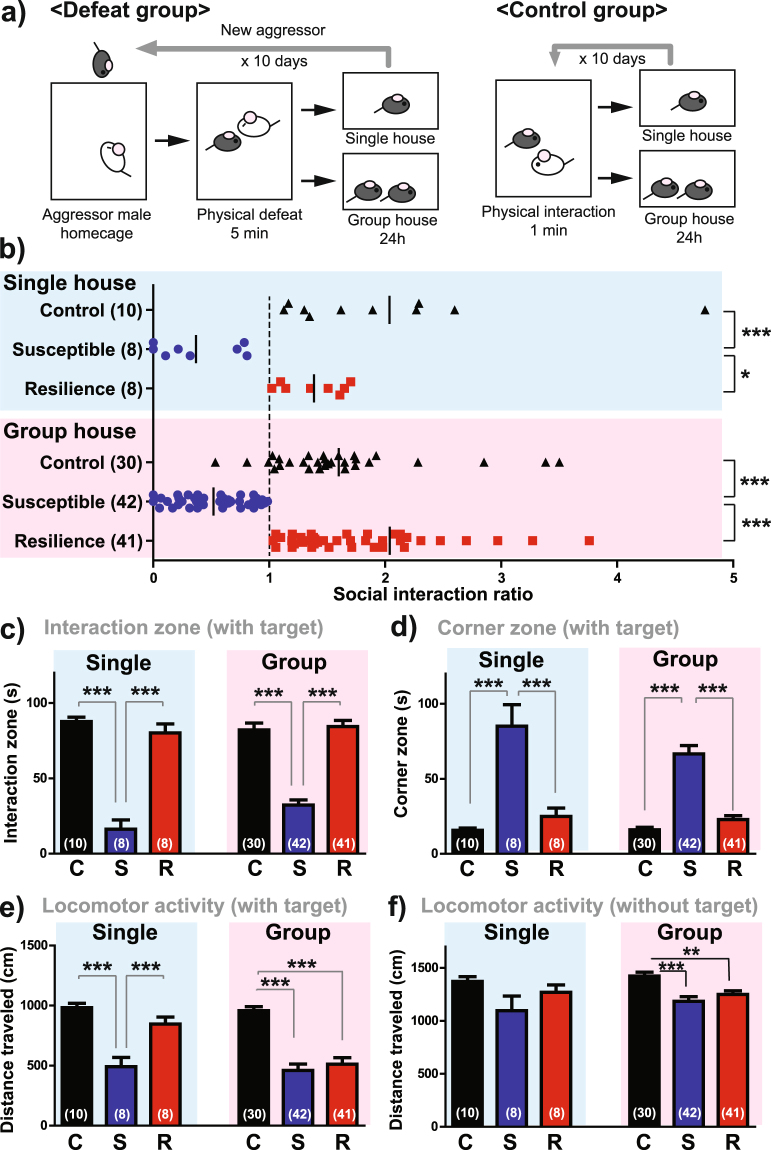
Adapted RSDS model without extended sensory contact produces robust social avoidance in a subset of female mice. (**a**) Experimental schematic of the RSDS model for female C57BL/6J mice without extended sensory contact. (**b**) Distribution of SI ratios in control and defeated females in single housed (top) and group housed (bottom) conditions. Time spent in the interaction zone (**c**), time spent in the corner zone (**d**) in the presence of novel male social target. Locomotor activity in the presence (**e**) and absence (**f**) of a social target. Data from the single-housed condition (blue) and group-housed condition (pink) are presented separately. C: control, S: susceptible, R: resilient. Data represent mean ± SEM. Number of animals is indicated in parentheses. *p < 0.05; **p < 0.01; ***p < 0.001 in Tukey’s *post hoc* test after One-way ANOVA.

To minimize the confounding effect of exposing females to mating-associated sensory signals following physical defeat, we performed RSDS without any post-defeat sensory contact. We conducted all aggressive encounters in a standard, static mouse cage with a clear Plexiglas top. Because defeat in these smaller cages tended to be more intense, we shortened the duration of defeat to 5 minutes in order to prevent significant injury. Following each defeat bout, we housed females either alone or together with another female (without divider) in a separate mouse cage until the next defeat episode (Fig. 3a). In both single-housed and group-housed condition, we found that about 50% of defeated females showed strong social avoidance (Fig. 3b). One-way ANOVA indicated a significant main effect of group on SI ratio for both single-housed (F(2,25) = 11.87, p = 0.0003) and group-housed conditions (F(2,112) = 28.82, p < 0.0001), in which susceptible females exhibited lower SI ratios than both control and resilient females. When the social target was present, susceptible females showed a strong reduction of time spent in the interaction zone (Single: F(2,25) = 61.09, p < 0.0001, Group: F(2,112) = 58.67, p < 0.0001; Fig. 3c), increased duration in corner zones (Single: F(2,25) = 20.65, p < 0.0001, Group: F(2,112) = 48.95, p < 0.0001; Fig. 3d), and reduced locomotor activity (Single: F(2,25) = 19.77, p < 0.0001, Group: F(2,112) = 25.72, p < 0.0001; Fig. 3e). In group-housed condition, both resilient and susceptible animals showed reduced locomotor activity compared to control females regardless of existence of target (Fig. 3e,f). In the absence of a social target, we observed no differences between groups in most behavioral indices, with the exception of a slight reduction in interaction zone duration in single-housed susceptible mice compared to resilient mice, but not control mice (F(2,25) = 3.575, p = 0.0445).

We found no difference in the amount of aggression experienced by susceptible and resilient females (Supplemental Fig. 4a). We also observed no effect of defeat on estrous cycle stage in susceptible or resilient females compared to controls (Supplemental Fig. 4b). Furthermore, 2-way ANOVA revealed no effect of estrous cycle stage on SI ratio or time spent in the interaction zone in control, susceptible and resilient females (Supplemental Fig. 4c‒e). However, there was a significant interaction of estrous cycle stage and defeat on time spent in the corner zone (F(2,55) = 7.358, p = 0.0015) and a trend toward an interaction of estrous cycle stage and defeat on time spent in the interaction zone (F(2,55) = 2.705, p = 0.0757), whereby, only within the susceptible group, diestrus females spent less time in the interaction zone and more time in the corner zones compared to estrus females (Supplemental Fig. 4d,e). There was no effect of estrous cycle stage on these indices in control or resilient groups. Also, there was no difference in the degree of wounding between resilient and susceptible animals (Supplemental Fig. 5).

In order to compare males and females using our RSDS paradigm, it is essential that our modified procedure for females is also effective in males. Accordingly, we tested male C57BL/6J mice with the same, sensory contact-lacking RSDS protocol using wild type CD-1 males as aggressors (Supplemental Fig. 6a). In contrast to females, the proportion of susceptible males was greater in the group-housed condition (36%) than single-housed condition (20%). Importantly there were no differences in the proportion of susceptible group-housed males (36%) versus susceptible group-housed females (36%) using this protocol. Susceptible males in both housing conditions exhibited lower SI ratios (Single: F(2,29) = 7.127, p = 0.0033, Group: F(2,27) = 7.523, p = 0.0028), spent less time in the interaction zone when the target was present (Single: F(2,29) = 9.070, p = 0.0010, Group: F(2,27) = 15.43, p < 0.0001) and spent more time in the corner zone when the target was present (Single: F(2,29) = 3.165, p = 0.0582, Group: F(2,27) = 5.975, p = 0.0076) compared to control and resilient males (Supplemental Fig. 6b‒d). We found no difference between groups in locomotor activity or body weight gain (Supplemental Fig. 6e,f).

In order to utilize this model to study sex differences in depression-like behavior, one must be sure that there are no differences in aggressive behaviors towards male versus female intruders. As shown in Supplemental Fig. 7, we do see a slight non-significant trend toward an increase in number of attack bouts and attack duration in aggressive interactions of DREAAD-injected ERα-Cre F1 aggressors toward males than toward females. Compared to CD-1 aggressors in traditional male aggressive behavior, DREAAD-injected ERα-Cre F1 aggressors attack with low latencies in both males and females (less than 10 seconds). Duration and frequency of attack bouts of DREAAD-injected ERα-Cre mouse toward female was close to the level of aggressive behavior of CD-1 aggressors toward male. In order to study sex differences, experimenters should be aware of this potential difference in selecting DREAAD-injected ERα-Cre F1 aggressors during the screening process. Experimenters should try to select aggressors that attack males and females equally to avoid using aggressors that exhibit a male attack bias.

### Behavioral and physiological profile of females exposed to adapted RSDS

**Figure 4:**
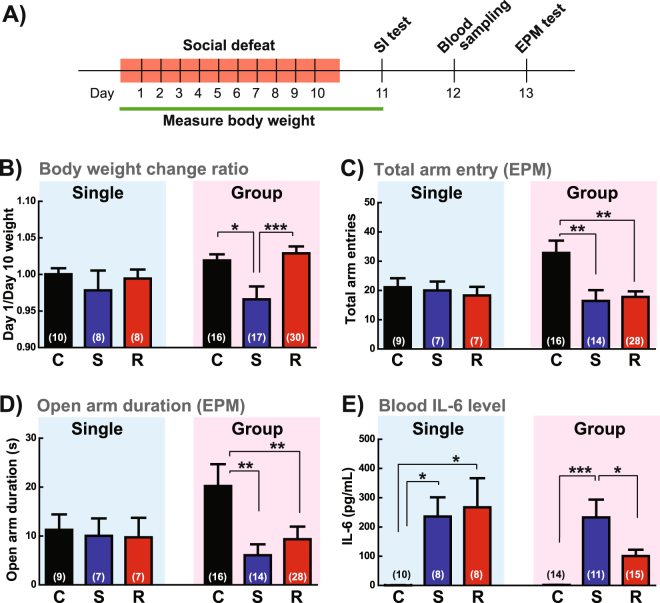
Behavioral and physiological profile of defeated females. (**A**) Time line of this experiment. EPM: elevated plus-maze. (**B**) Change of body weight from Day 1 (before defeat) to Day 10 of RSDS. (**C**) Total arm entry and (**D**) time spent in the open arm in the EPM. (**E**) Serum IL-6 level 24 hours after the SI test. Data from single-housed condition (blue) and group-housed condition (pink) were presented separately. C: control, S: susceptible, R: resilient. Data represented mean ± SEM. Number of animals is indicated in parentheses. *p < 0.05; **p < 0.01; ***p < 0.001 in Tukey’s *post hoc* test after One-way ANOVA.

To further characterize the effect of RSDS on female group-housed mice, we measured body weight during the adapted RSDS procedure, performed the elevated plus maze test to examine anxiety-like behaviors, and assayed blood levels of interleukin 6 (IL-6) previously implicated in male susceptibility to RSDS^22^ (Fig. 4A). We observed a significant main effect of defeat on body weight gain (F(2,64) = 7.642, p = 0.0011; Fig. 4B), with susceptible females exhibiting a reduction of body weight compared to control and resilient females. We also observed a significant main effect of stress on total arm entries (F(2,57) = 8.045, p = 0.0009; Fig. 4C) and duration in the open-arm in the elevated plus maze (F(2,57) = 4.508, p = 0.0154; Fig. 4D) in defeated mice compared to controls; however, there were no differences between susceptible and resilient mice in any of these measurements. This pattern of reduced exploratory behavior was also evident in the SI test when a social target was absent from the wire enclosure (essentially an open-field test) in both susceptible and resilient females compared to controls (Fig. 3f). These behavioral findings are consistent with those previously reported in male C57BL/6J mice following standard RSDS^18^.

In contrast, there was no effect of social defeat in single-housed females on any of the above measures. This finding was largely due to a pro-depressant and anxiogenic effect of social isolation in control females that caused a ceiling effect (Fig. 4B‒D).

We next tested whether RSDS affects peripheral inflammatory responses in females as we have shown such responses to be an important determinant of stress susceptibility in male mice and in humans^22,38^. We measured blood IL-6 levels of females in both the group-housed and single-housed conditions 48 hours after the last defeat bout. As in males, we found that RSDS strongly increased IL-6 in the blood of group-housed susceptible mice, whereas resilient and control mice showed low to undetectable levels of IL-6 (F(2,39) = 12.13, p < 0.0001; Fig. 4E). However, in the single-housed condition, both susceptible and resilient females showed elevated blood levels of IL-6 (F(2,25) = 5.681, p = 0.0099) after RSDS, which again suggests that single housing adds a significant stressor to the female RSDS protocol. Overall, our data indicate that RSDS in the group-housed condition most consistently reproduces the behavioral and physiological endpoints observed in male mice following standard RSDS.

## Discussion

Despite higher lifetime prevalence rates of depression and anxiety disorders in women, the majority of rodent studies examining the mechanisms of mood disorders have been conducted exclusively in males. In this study, we established a female model of RSDS by using DREADD-based activation of the hypothalamic attack locus VMHvl to induce aggression in male resident mice toward female intruders. CNO-induced aggression toward female mice altered key behavioral and physiological properties of defeated females, consistent with previously validated results in male mice. In addition, as observed in males^17,18^, we found subpopulations of defeated females to be stress-susceptible or stress-resilient following RSDS. Susceptible females showed social avoidance, reduced body weight, and increased IL-6 compared to control and resilient females. By contrast, susceptible and resilient females both showed increased anxiety-like behaviors compared to control females, however, resilient females did not exhibit social avoidance or weight loss. These patterns of behavioral abnormalities closely resemble susceptibility and resilience in male mice after RSDS^12,18^.

Aside from inducing aggressive behavior with DREADDs, we made several additional modifications to the RSDS paradigm to adapt the model to female mice. During our initial experiments, we found that overnight housing across a Plexglas partition from a formerly aggressive male had a buffering effect on the development of depression- and anxiety-like behaviors in females. We therefore tested two housing conditions (group housed with other females or single housed) to mitigate these buffering effects. However, we found  that social isolation by itself in females caused increased expression of depression- and anxiety-like phenotypes in the absence of RSDS compared to group-housed controls. This finding is consistent with work showing that female mice are more sensitive to social isolation than males during the preadolescent period^39^. In addition, male rats, which tend to be more sensitive to social isolation than male mice, exhibit enhanced expression of anxiety-like behaviors and hypothalamic-pituitary-adrenal (HPA) activity following social defeat^40^.

In our current study, we found that the group-housed condition is most appropriate for investigation of individual differences in stress susceptibility, as susceptible females in this condition exhibited behavioral and physiological outcomes consistent with those reported in males exposed to RSDS^18,22^—strong social avoidance, significant reduction of body weight and elevated peripheral IL-6. Unlike in females, the single-housed condition did not seem to compound the stressful effect of RSDS in C57BL/6J male mice as social isolation during the course of 10-day defeat did not increase the percentage of susceptible male mice. Thus, our data show that female mice are more vulnerable than males to brief periods of social isolation whereas sensory contact enhances the defeat phenotype in male mice.

It is generally thought that female mice are more sensitive to stress than males. However, this is highly dependent upon the type of stress and the behavioral indices and species being investigated. For example, following repeated variable stress, which is a combination of mild footshock, tail suspension and restraint stress, female mice develop depressive- and anxiety-like behaviors more rapidly than males^11,41^. In contrast, in the learned helplessness model, female rats^42^ and C57BL/6 mice (but not 129SvEv mice)^43^ are less sensitive to uncontrollable stress and show active avoidance behaviors even after repeated uncontrollable footshock stress.

In social defeat stress models, there are some species where both females and males exhibit aggression toward a same-sex intruder, including the Syrian hamster (*Mesocricetus auratus*) and California mouse (*Peromyscus californicus*)^44,45^. A single social defeat experience from same sex conspecifics induces learned submissive behaviors in all male Syrian hamsters, while only a subset of females exhibit similar behaviors^46,47^. Conversely, female, but not male, California mice display social avoidance behavior following social defeat by a same sex conspecific^48^. Postpartum female rats exhibit aggression toward female intruders, and such maternal aggression induces several depression-like behaviors including reduced body weight and disruption of estrous cycle^49,50^ (but see^51^).

Other models have lesioned the mediobasal hypothalamus of female rats to induce female aggressive behavior, however, these models do not induce the change in body weight or corticosterone that is observed in male rats^52^. While all of the above mentioned models have value for better understanding female stress responses, the model described here allows us to exploit key transgenic and knockout technologies to test mechanistic hypotheses related to stress-induced depression- and anxiety-like behavior. In our female defeat model, it is important to note that use of a male aggressor to socially defeat a female intruder (opposite sex), may be qualitatively different from male social defeat stress (inter-male). However, because male-female aggression has a major impact on women’s mental health, we feel that this model fills a critical gap in our ability to understand biological mechanisms of susceptibility and resilience to social stress in female rodents.

In summary, we have established a female social defeat model in C57BL/6J mice that, consistent with the standard RSDS model in males, produces individual differences in behavioral susceptibility to social stress, also reflected in peripheral IL-6 levels, showing at least some overlap in biological mechanisms between the sexes. This new female social defeat model will thus allow us to expand upon previously published reports in males through the study of sex differences in depression- and anxiety-related biological pathways. Such studies may ultimately enable us to identify sex-related mechanisms of stress susceptibility and antidepressant efficacy, and to better formulate clinical studies for sex-specific therapeutic approaches.

## Methods

### Animals

7 week old C57BL/6J female mice (The Jackson Laboratory, Bar Harbor, Maine) were group housed (5 mice per cage) in standard mouse cages (28.5 cm (w) × 18.5 cm (d) × 12.5 cm (h)) for a week before the social defeat procedure. Body weights were taken one day before the beginning of repeated social defeat to assign defeat and control groups to have similar average starting body weight.

For aggressors, we used CD-1 male mice and Esr1 (ERα)-Cre mice. CD-1 male mice were sexually experienced breeders at least 4 months of age (Charles River Laboratories). ERα-Cre mice with a C57BL/6J background^34^ were purchased from the Jackson laboratory and crossed with CD-1 females in the breeding facility at the Icahn School of Medicine at Mount Sinai (ISMMS) to obtain F1 offspring. F1 offspring were group housed with their siblings until the VMHvl surgery, which was conducted when the mice were about 8 weeks old. After the surgery, all males were housed individually throughout the experiment. Mouse procedures were performed in accordance with the National Institute of Health Guide for Care and Use of Laboratory animals and all procedures were approved by the ISMMS Animal Care and Use Committee (approval number LA10-00266).

### DREADD expression in the VMHvl of aggressor males

AAV vectors encoding a non-conditional Gq-DREADD^53,54^ (AAV2-hSyn-HA-hM3D(Gq)-IRES-mCitrine; University of North Carolina, Chapel Hill, NC) or a conditional AAV vector (rAAV2/hSyn-DIO-hM3D(Gq)-mcherry; University of North Carolina, Chapel Hill, NC) were used in wild type CD-1 or ERα-Cre F1 mice, respectively, to target the VMHvl. Male mice were anesthetized with a cocktail of ketamine HCl (100 mg/kg) and xylazine (10 mg/kg) injected intraperitoneally (i.p.). A 33 gauge needle attached to a glass Hamilton syringe was stereotaxically inserted into the VMHvl (AP, −1.5; ML, ± 0.7; DV, −5.7 mm from bregma) bilaterally as calculated from the mouse brain atlas (Paxinos and Franklin). The AAV vector was infused in a volume of 0.3 ul/side over 3 min, and the needle was left in place for 5 min after the injection. Two-weeks after the AAV injection, these mice were screened for aggressive behavior toward females. Thirty-five min before the screening, each male mouse was intraperitoneally injected with CNO (1 mg/kg). Following this incubation period, a C57BL/6J female was introduced into the male’s homecage for 5 min. This screening was repeated one week later, and male mice that showed aggressive behavior toward female in both screenings were used for the subsequent social defeat procedure as aggressors. If the male showed aggressive behavior only once during these initial screenings, third screening session was performed to select males that attacked during at least 2 out of 3 sessions.

### Conventional repeated social defeat stress (RSDS) procedure

The RSDS protocol was similar to previously published protocols in males with the following modifications^12^. A standard hamster cage (26.7 cm (w) × 48.3 cm (d) × 15.2 cm (h)) containing hard woodchip bedding was divided into two compartments by two clear perforated Plexiglas dividers (0.6 cm (thickness) × 45.7 cm (d) × 15.2 cm (h)) inserted in the middle of the cage. Two dividers between the aggressor and females were necessary because females tended to cross a single divider to the aggressor male compartment during the evening when CNO-induced aggression subsided. The hamster cage was covered by a steel-wire top, and food and water were supplied *ad libitum* to both compartments. One day before the first social defeat, aggressor CD-1 males were housed in one side of the hamster cage for habituation.

On the first day of RSDS, aggressors were injected with CNO (1 mg/kg, i.p.) 35 min before the encounter. A C57BL/6J female was confined to the aggressor’s side for a total of 10 min following an initial attack. Then, females were transferred to the opposite compartment of the same cage. If aggressor males exhibited any type of sexual behaviors such as mounting behavior or thrusting, we immediately removed the female and placed her in another aggressor’s cage. If the second aggressor also did not show any aggressive behavior, we ended the encounter for the day. Following successful aggressive interaction with a male, the female was housed on the opposite side of the hamster cage from the aggressor for the remainder of the 24 hour period. This replicates the psychosensory exposure period in the male RSDS protocol^12^, where the defeated mouse can see, smell and hear the aggressor in the absence of physical contact, and is thought to produce psychological stress. The following day, aggressors were again injected with CNO (1 mg/kg, i.p.) 35 min before the encounter, and the procedure was repeated for 10 days total. Animals were then individually housed in standard mouse cage after the last physical defeat session. Mouse body weight was measured every day before the physical defeat stress.

Control mice were placed in a standard mouse cage containing hard woodchip bedding divided into two compartments by a clear perforated Plexiglas divider. Due to the possibility that exposure to male pheromones could change the behavior of females (Supplemental Fig. 3), we housed a control C57BL/6J female with a CD-1 male to be as consistent as possible with the defeat group. The female was allowed to interact with the CD-1 male (without CNO injection) by removing divider for 1 min every day in their homecage. The control interaction was restricted to 1 min to prevent the initiation of sexual behavior. After the 10 days of RSDS, both defeated and control females were housed individually in new standard mouse cage.

### Adapted RSDS without extended sensory contact

Male aggressors were housed individually in standard mouse cages (28.5 cm (w) × 18.5 cm (d) × 12.5 cm (h)) with hard woodchip bedding. Test females were housed either individually or paired with another female (defeated female pair-housed with defeated female) in the standard mouse cage without divider. Similar to the conventional RSDS method, aggressors were injected with CNO (1 mg/kg, i.p.) 35 min before the encounter. To better observe aggressors’ behavior, the wire mesh cage top was removed and replaced with a clear Plexiglas top just prior to the aggressive encounter. A C57BL/6J female was introduced into the aggressor’s cage for 5 min after the first attack. Because defeat in these smaller cages tended to be more intense, we shortened the duration of defeat from 10 min to 5 min in order to reduce significant injury. If females were not physically attacked by an aggressor, they were removed to another aggressor’s cage for 1 more session. After the physical defeat, the female was returned to her homecage. On the following day, the female was introduced to a novel aggressor’s cage to experience 5 min of physical defeat stress and this procedure was repeated for a total of 10 days. Body weight was measured every day throughout the physical defeat stress. Control females were housed either individually or two per cage together in a standard mouse cage without divider. Each control female was placed in the homecage of an ERα-Cre F1 control male (without CNO injection) for a 1 min period of interaction on each of the ten days of RSDS. After the final RSDS or control interaction session, both defeated and control females were housed individually in new standard mouse cages.

### Social interaction (SI) test

The SI test was conducted one day after the last defeat session under exactly the same test conditions described for males in previous studies^12^. A wire-mesh enclosure (10 cm (w) × 6.5 cm (d) × 42 cm (h); Nationwide Plastics) was placed at one end of the white plastic open field (42 cm (w) × 42 cm (d) × 42 cm (h); Nationwide Plastics). A CCD camera was placed above the open field, and the video data was sent to a computer and analyzed by video-tracking software (Ethovision 3.0; Noldus Information Technology). Animals were acclimated to the test suite for 1 hour before the test. The test consisted of two 150-sec phases; a “target absent” phase and a “target present” phase. During the “target absent” phase (phase 1), no social stimulus was placed in the wire-mesh enclosure. During the “target present” phase (phase 2), a novel aggressor male was placed in the wire-mesh enclosure. Male mice were chosen such that they were novel but had the same genetic background as aggressors used for the RSDS procedure. Social target mice with or without CNO injection 30 min before the test were used. Social avoidance did not differ in groups exposed to aggressors with and without CNO prior to SI. In each phase, a C57BL/6J female was introduced into the side of the open field opposite the wire-mesh enclosure, and her exploratory behavior was automatically tracked for 150 sec by video-tracking software. Between phase 1 and 2, the C57BL/6J female mouse was returned to her homecage and for an approximately 30 second interval. Within the Ethovision program, an interaction zone (an 8 cm wide corridor surrounding the wire-mesh enclosure) and a corner zone (two 9 × 9 cm squares in the corners of the field opposite the wire-mesh enclosure) were defined within the open field, and the time spent by the test animal in those zones, as well as the total distance traveled in the open field were calculated. At the end of each test, both the C57BL/6J test female and the social target male mouse were returned to their homecages, and the test apparatus was cleaned with a quatricide solution. All behavioral tests were conducted under red light conditions.

### Estrous cycle monitoring

Vaginal cellular samples were collected immediately after the SI test by gently pipetting 10 µL of sterile saline into the vagina and dispensing the vaginal sample onto a glass slide. Samples were air dried and stained with Toludine blue^11^. Cycle stage was determined by visual inspection under a light microscope.

### Serum sampling and IL-6 measurement

Twenty-four hours after the SI test, blood samples were collected from the submandibular vein into protein Lobind tubes (Eppendorf). Whole blood was kept at room temperature for 1 hour prior to centrifugation for 15 min at 956 × g at 4 °C. Serum was collected and stored at −80 °C until the protein assay. Serum IL-6 levels in control and defeated animals were measured by Enzyme Linked Immunosorbent Assay (ELISA, Mouse IL-6 ELISA Kit, Cat#55950, BD OptEIA^TM^, BD Biosciences, USA) according to the manufacturer’s instructions. All samples were measured in duplicate.

### Elevated plus maze

Female mice were acclimated to the testing room for 1 hour before testing. The elevated plus maze apparatus consisted of black Plexiglas with two open arms (12 cm (w) × 50 cm (d) × 0.5 cm (h)) and two closed arms (12 cm (w) × 50 cm (d) × 40 cm (h)) placed 1 m above the floor. A CCD camera was placed on the ceiling of the testing room, and the video data was sent to a computer and analyzed by video-tracking software (Ethovision 3.0; Noldus Information Technology). The animal was placed in the center of the elevated plus maze, and her exploratory behavior was analyzed for 5 min. From the tracking data, total time spent in the open arm, total time spent in the closed arm, and total distance traveled were calculated.

### Statistical analysis

GraphPad Prism 5 software was used for statistical analysis. Unpaired t-tests were used to compare two groups, and one-way ANOVA was conducted to compare resilient, susceptible, and control females followed by Tukey’s multiple comparison *post hoc* tests. Two-way ANOVA was conducted to examine the effect of estrous cycle in susceptible, resilient, and control females followed by Tukey’s multiple comparison *post hoc* test. To analyze whether the distribution of SI ratio in defeated females followed a normal distribution, we used the Kolmogorov-Smirnov Goodness (KS) Test.

### Availability of materials and data

The datasets generated during the current study are available from the corresponding author on reasonable request.

## Electronic supplementary material

Supplementary information

Supplemental Video 1

Supplemental Video 2
