# Supplementary material for: Establishment of a repeated social defeat stress model in female mice
Source: Sci Rep. 2017 Oct 9;7:12838. doi: 10.1038/s41598-017-12811-8 (PMC5634448; doi:10.1038/s41598-017-12811-8)
Supplement: Supplementary file 1 — Supplementary information [file 41598_2017_12811_MOESM1_ESM.pdf]

Supplementary Information

Establishment of a repeated social defeat stress model in female mice

Aki Takahashi, Jia-Ru Chung, Song Zhang, Hongxing Zhang, Yael Grossman,
Hossein Aleyasin, Meghan E. Flanigan, Madeline L. Pfau, Caroline Menard, Dani Dumitriu,
Georgia E. Hodes, Bruce S. McEwen, Eric J. Nestler, Ming-Hu Han, Scott J. Russo

Contents

Supplemental Figure 1. SI phenotypes of females exposed to conventional RSDS with ERα-Cre aggressors.

Supplemental Figure 2. Extended characterization of conventionally defeated females.

Supplemental Figure 3. Comparison of control females housed with a CD-1 male and control females housed with a C57BL/6 (B6) female.

Supplemental Figure 4. Extended characterization of females exposed to adapted RSDS.

Supplemental Figure 5. Amount of wounds received during RSDS was not different between resilient and susceptible females.

Supplemental Figure 6. Behavioral analysis of C57BL/6J male mice exposed to the adapted RSDS model without extended sensory contact.

Supplemental Figure 7. Aggressive behavior of ERα-Cre aggressor males toward male and female C57BL/6J intruders.

Supplemental Methods

Supplemental Reference

Supplemental Video 1. Aggressive behavior of CD-1 aggressor male after CNO injection in the conventional RSDS paradigm.

Supplemental Video 2. Aggressive behavior of ERα-Cre aggressor male mice after CNO injection in the adapted RSDS paradigm without social contact.

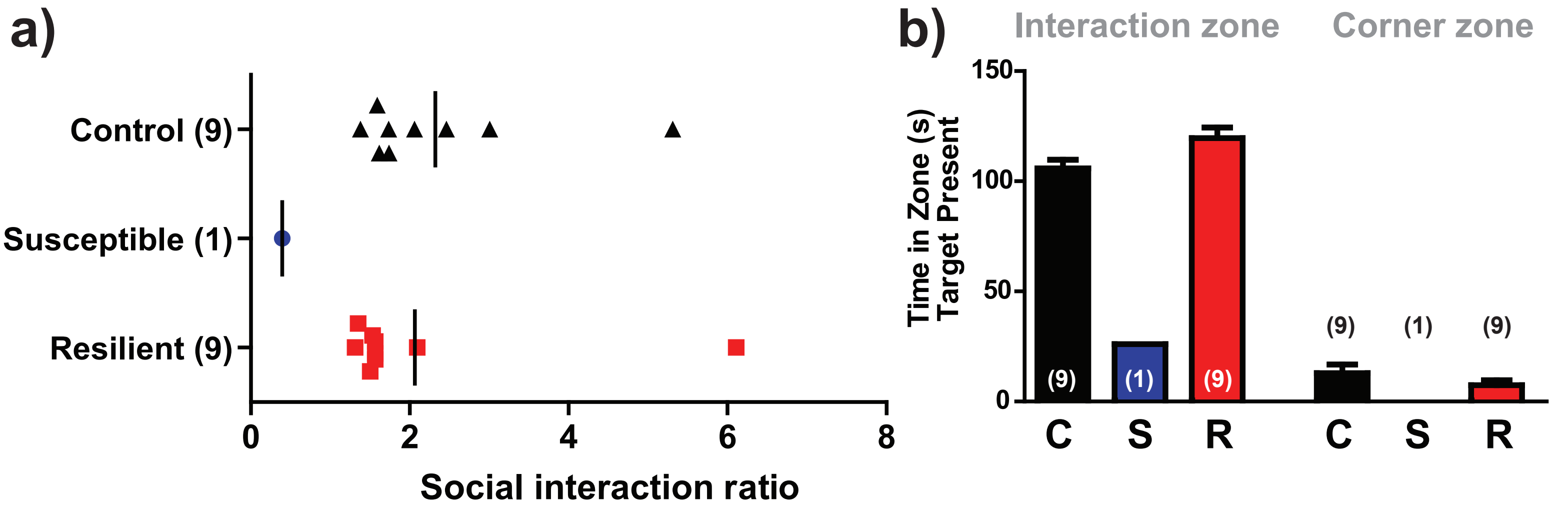

Supplemental Figure 1. SI phenotypes of females exposed to conventional RSDS with ERα-Cre aggressors. (a) Distribution of SI ratios in control and defeated female C57BL/6J mice. (b) Time spent in the interaction zone and corner zone when the target was present. C: control, S: susceptible, R: resilient. Data represented mean ± SEM. Number of animals is indicated in parentheses.

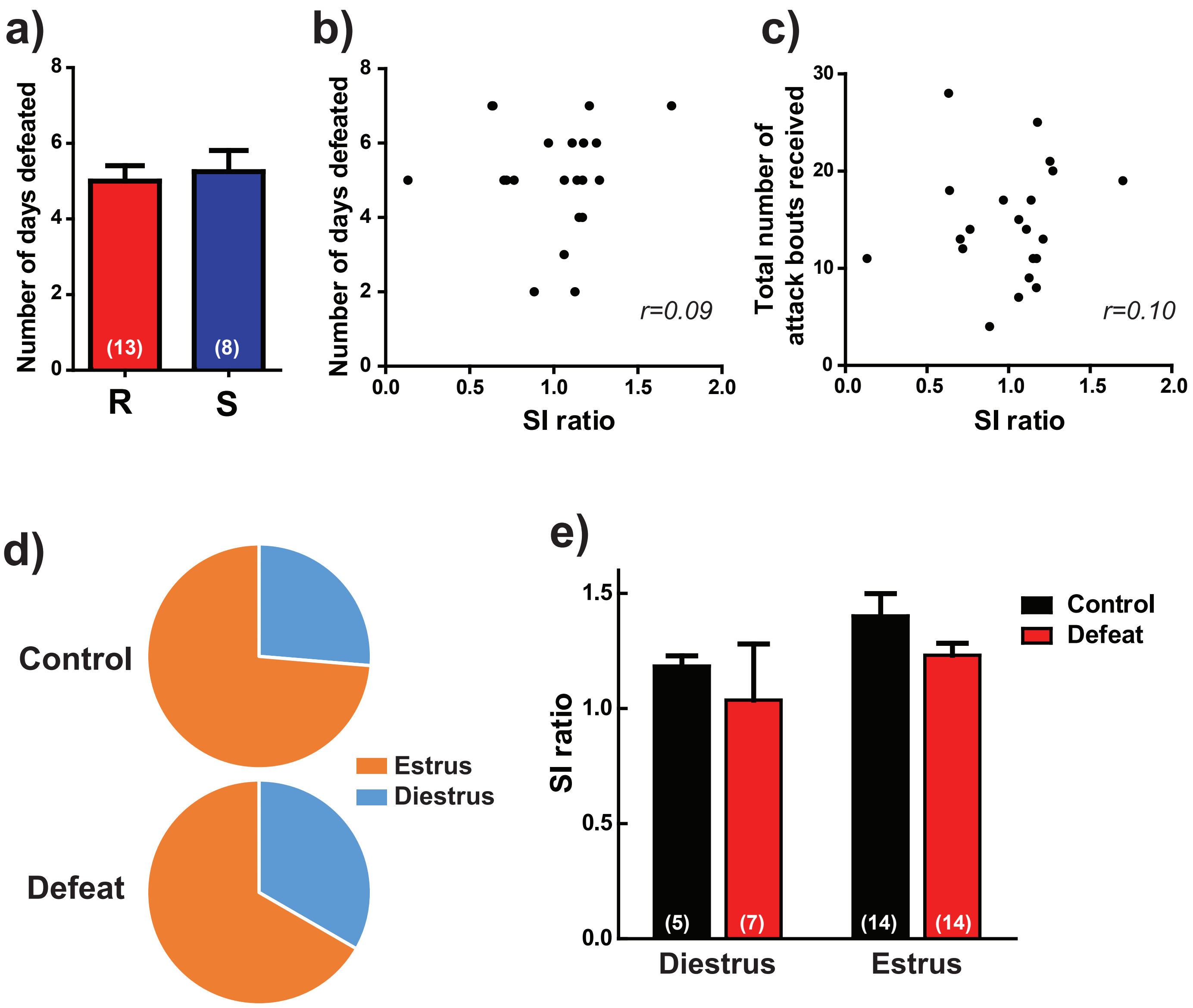

Supplemental Figure 2. Extended characterization of conventionally defeated females. (a) The number of days that female C57BL/6J mice received aggressive attacks from male CD-1 aggressors does not differ between resilient and susceptible females. There is no correlation between SI ratio and (b) the number of days defeated or (c) the frequency of attack bouts during the 10-day RSDS. (d) Estrous cycle stage of control and defeated females at the point of SI testing. (e) SI ratio in diestrus and estrus females. Data represent mean ± SEM. Number of animals is indicated in parentheses.

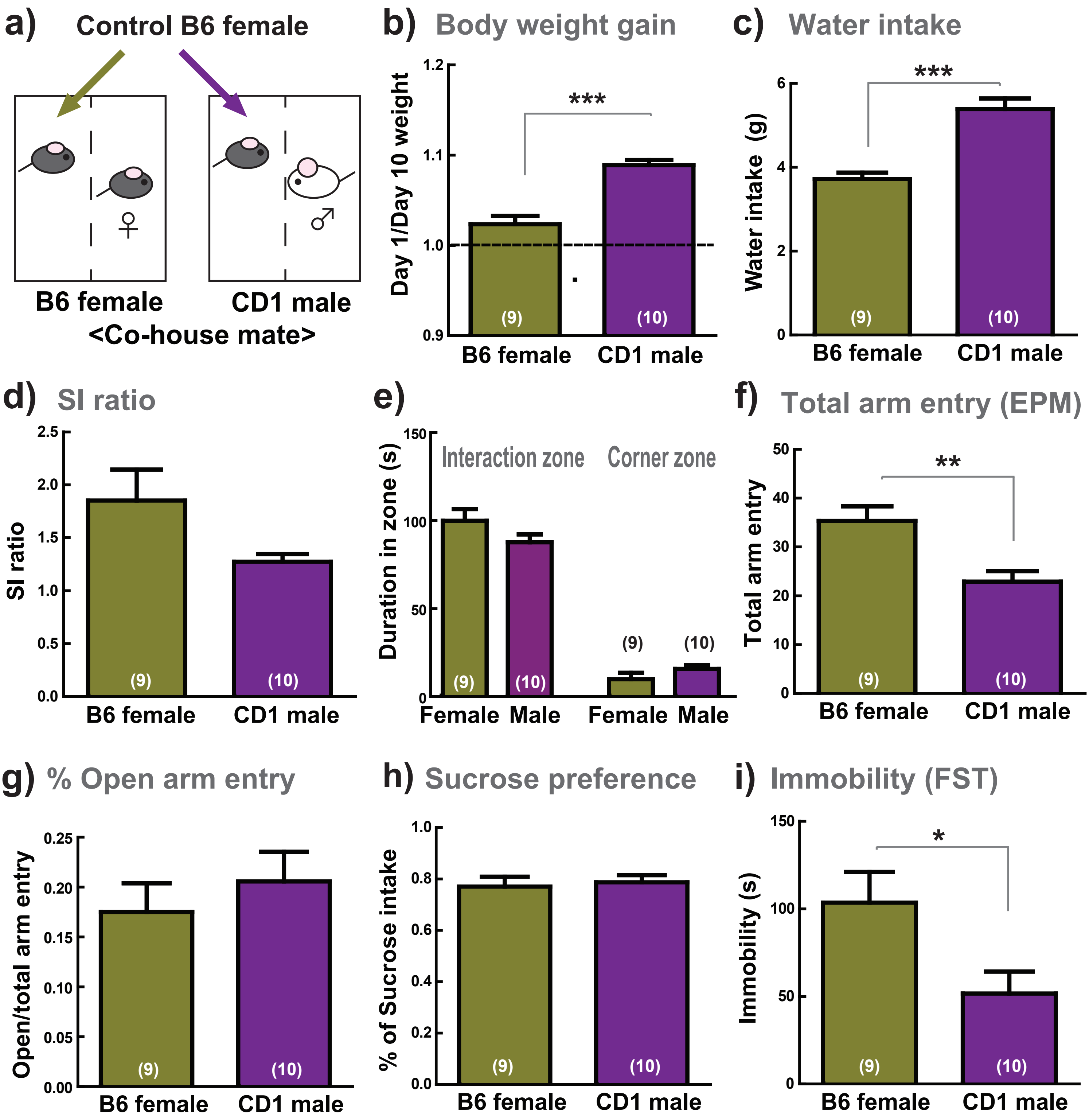

Supplemental Figure 3. Comparison of control females housed with a CD-1 male and control females housed with a C57BL/6 (B6) female. (a) Experimental schematic. A test C57BL/6J female was housed with either a CD-1 male or another C57BL/6J female across a perforated Plexiglas divider for 10 days. (b) Change of body weight from Day 1 to Day 10. (c) Amount of water intake from day 10 to day 11. (d) SI ratio from SI test using a male CD-1 social target. (e) Time spent in the interaction zone and the corner zone. (f) Total arm entry and (g) percentage of open arm exploration in the elevated plus maze (EPM). (h) Preference of 1% sucrose intake in two-bottle choice test. (i) Duration of immobility in the forced swim test (FST). Data represent mean ± SEM. Number of animals is indicated in parentheses. * $p<0.05$; ** $p<0.01$; *** $p<0.001$, unpaired t-tests.

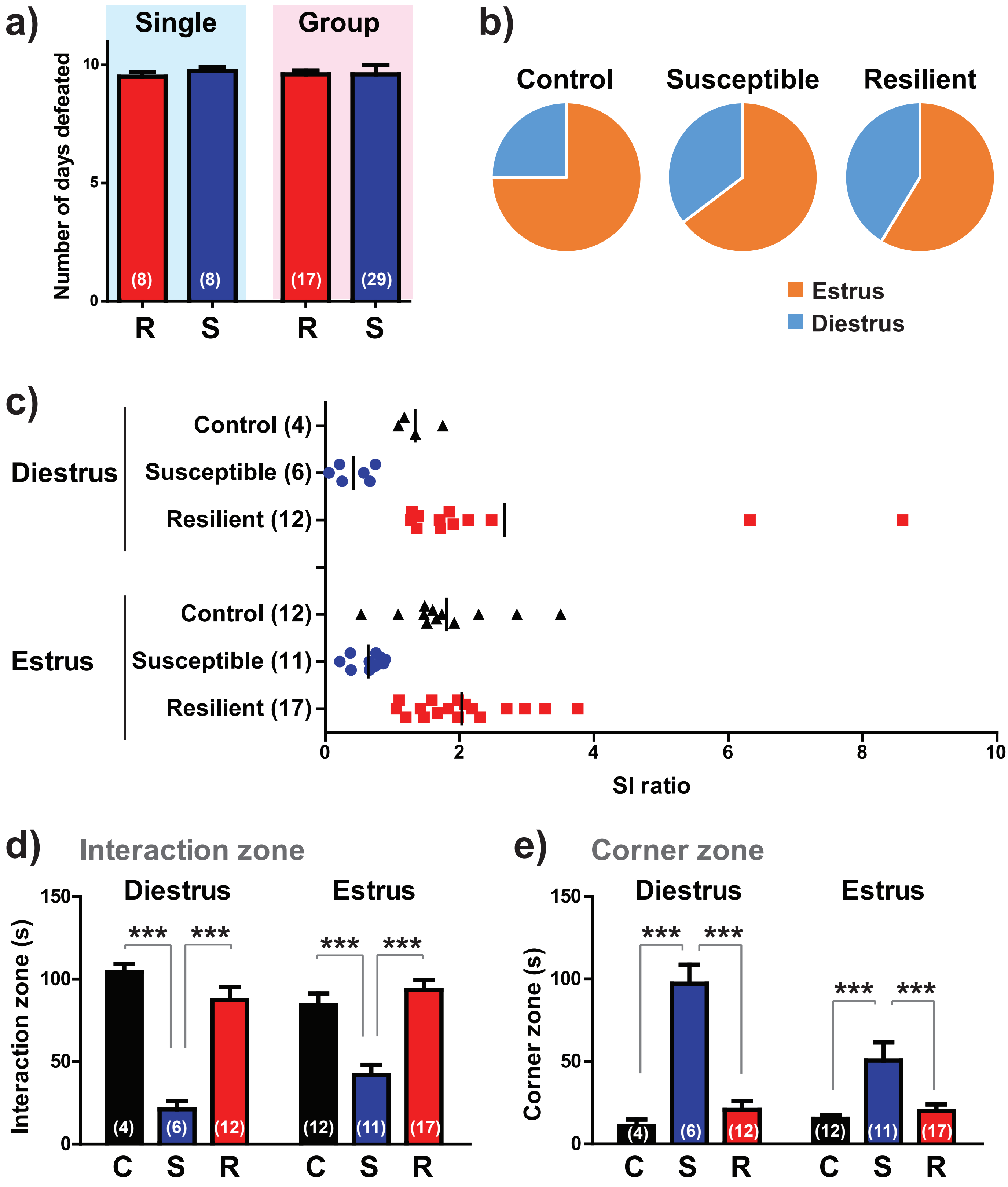

Supplemental Figure 4. Extended characterization of females exposed to adapted RSDS. (a) The number of days that female C57BL/6J mice received an aggressive attack from male aggressors does not differ between resilient and susceptible females in single- and group-housed condition. (b) Estrous cycle of control, susceptible and resilient animals at the point of SI testing. Only group-housed female data is presented. (c) Distribution of SI ratios, (d) time spent in the interaction zone and (e) time spent in the corner zone in diestrus and estrus females. Data represent mean ± SEM. Number of animals is indicated in parentheses. * $p<0.05$; ** $p<0.01$; *** $p<0.001$, Tukey’s post hoc test after Two-way ANOVA.

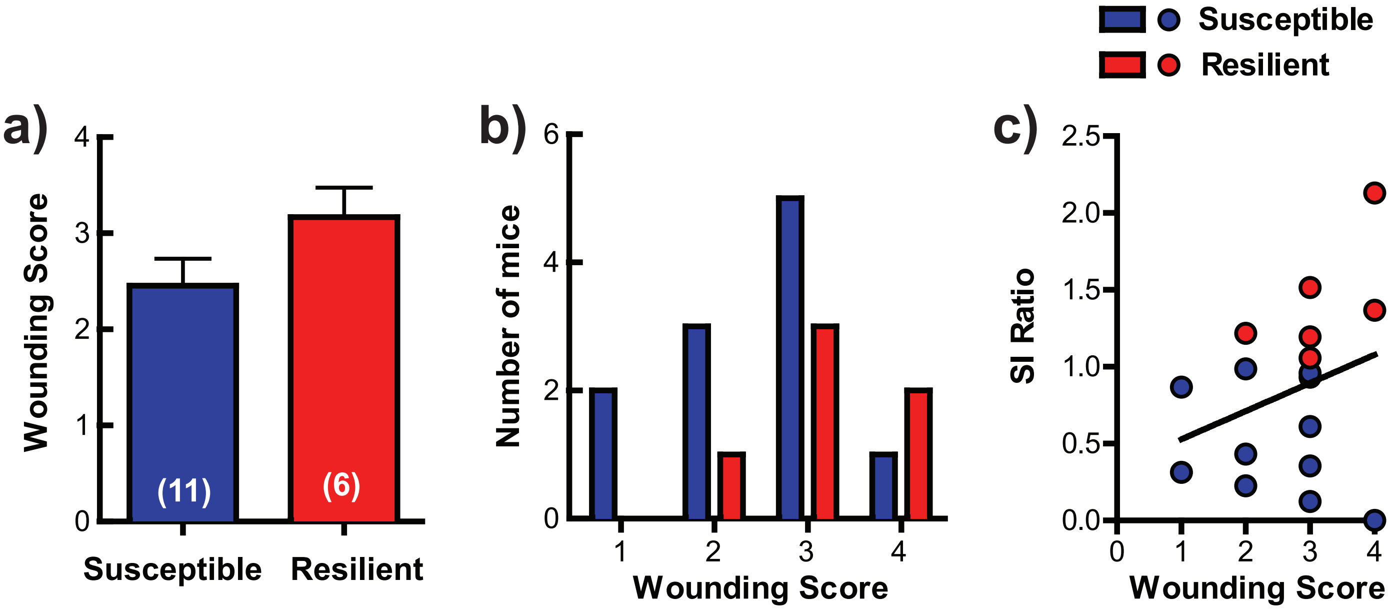

Supplemental Figure 5. Amount of wounds received during RSDS was not different between resilient and susceptible females. (a) Average wounding score of susceptible and resilient females. Data represented mean ± SEM. Number of animals is indicated in parentheses. (b) Distribution of the number of susceptible and resilient animals in each wounding score. (c) Correlation between wounding score and SI ratio in defeated females. No correlation was observed ($r^2 = 0.08917$, $p = 0.2443$).

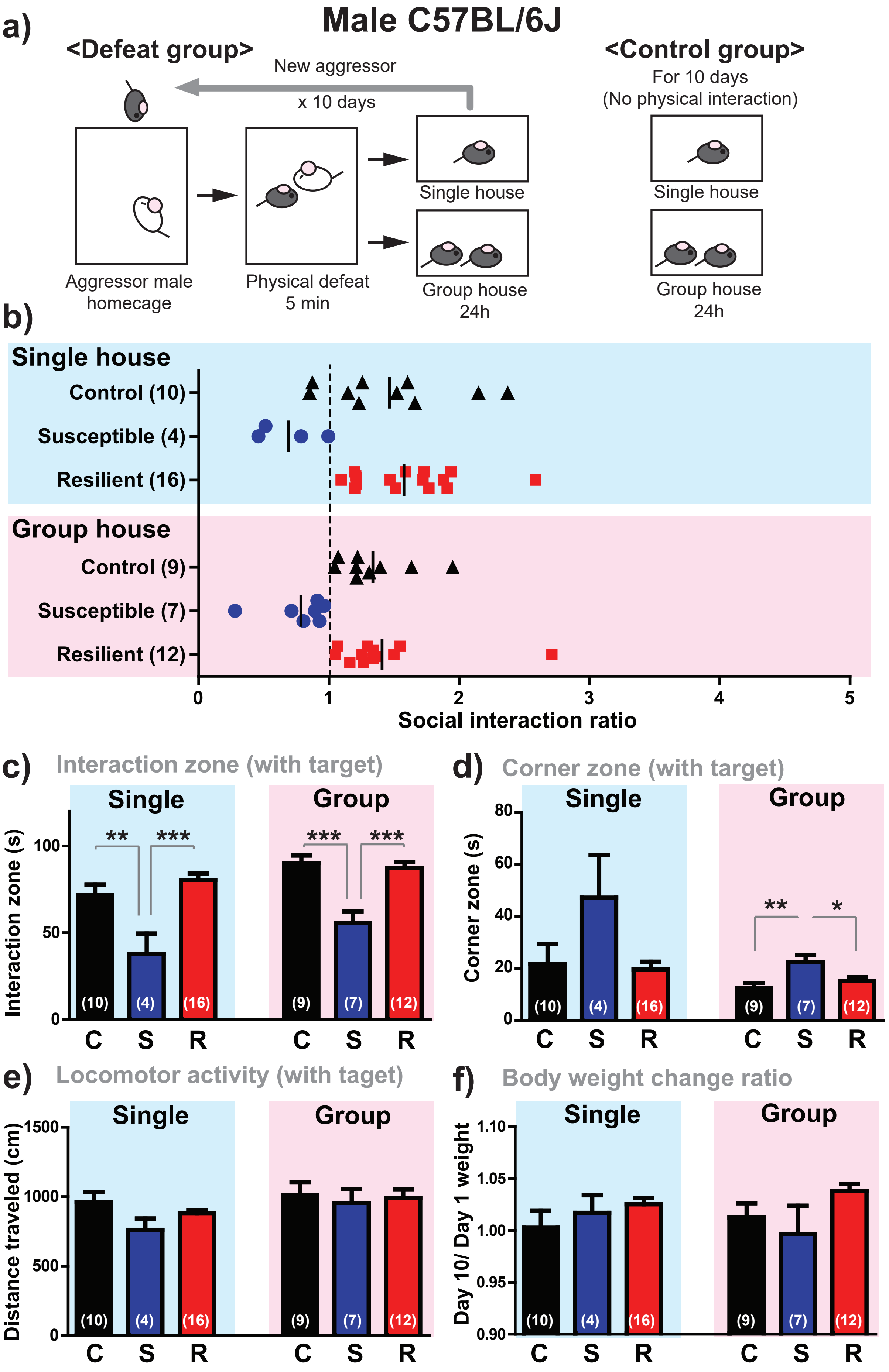

Supplemental Figure 6. Behavioral analysis of C57BL/6J male mice exposed to the adapted RSDS model without extended sensory contact. (a) Experimental schematic of RSDS model without sensory contact. Each day for 10 days, males were exposed to a 5 minute bout of physical aggression by wild-type CD-1 aggressors (not CNO-induced, usual territorial aggression) and then returned to their homecages, where they were housed alone (single-housed) or together with another defeated male (group-housed) for 24 hours. Control males were single- or group-housed for 10 days, during which they were not exposed to any physical interactions with CD-1 male. (b) Distribution of SI ratios in control and defeated males from single-housed and group-housed conditions. (c) Time spent in the interaction zone and (d) corner zone, and (e) locomotor activity in the presence of the novelCD-1 social target. (f) Change of body weight from Day 1 (before defeat) to Day 10 of RSDS. Data from single-housed condition (blue) and group-housed condition (pink) are presented separately. C: control, S: susceptible, R: resilience. Data represent mean ± SEM. Number of animals is indicated in parenthesis. * $p<0.05$; ** $p<0.01$; *** $p<0.001$, Tukey's post hoc test after One-way ANOVA.

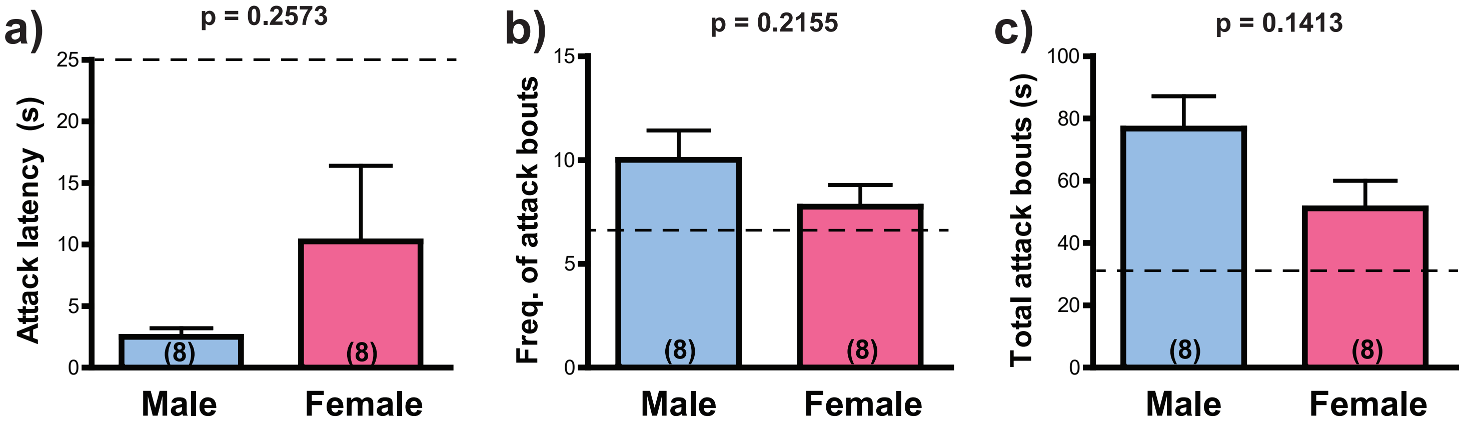

Supplemental Figure 7. Aggressive behavior of ERα-Cre aggressor males toward male and female C57BL/6J intruders. (a) Attack latency, (b) frequency of attack bouts, and (c) total duration of attack bouts of ERα-Cre aggressors toward male and female intruders. Dotted lines indicate the level of aggressive behavior of CD-1 aggressors toward male C57BL/6J intruders (Data from Golden et al 2016, aggressor animals' Day 3 aggression). Data represent mean ± SEM. Number of animals is indicated in parenthesis. Paired *t*-test was conducted to compare aggressive behavior of ERα-Cre aggressors toward male and female.

SUPPLEMENTAL METHODS

Quantification of wounding in the defeated animals

Body wounding was assessed at the point of sacrifice. Wounds were counted and/or measured (coverage area) for four bodily regions: the lower (1) and upper (2) regions of the back, the abdomen and legs (3), and the tail (4). Wound scores represent a composite score combining subscores for all 4 of these regions. Wound scores ranged from 0-4 for each region, with higher scores indicating greater wound severity. The maximum composite wound score was 16.

Adapted RSDS without extended sensory contact in male C57BL/6J mice

Male CD-1 aggressors were housed individually in standard mouse cages (28.5 cm (w) x 18.5 cm (d) x 12.5 cm (h)) with hard woodchip bedding. Test males (C57BL/6J) were housed in pair with another male in the standard mouse cage. Right before the aggressive encounter, the wire mesh cage top of aggressor's homecage was removed and replaced with a clear Plexiglas top. A C57BL/6J male was introduced into the aggressor's cage for 5 min after the first attack. If males were not physically attacked by an aggressor, they were removed to another aggressor's cage for 1 more session. After the physical defeat, the male was returned to his homecage. On the following day, the male was introduced to a novel aggressor's cage to experience 5 min of physical defeat stress and this procedure was repeated for a total of 10 days. Body weight was measured every day throughout the physical defeat stress. Control males were housed two per cage together in a standard mouse cage. After the final RSDS or control interaction session, both defeated and control females were housed individually in new standard mouse cages.

Quantification of aggressive behavior of ERa-Cre aggressors toward male and female C57BL/6J mice.

Eight ERα-Cre F1 aggressor males that were used in this study were tested their aggressive behavior toward male and female C57BL/6J mice. Three-min resident-intruder test was conducted for 2 days to quantify aggressive behavior of ERα-Cre F1 aggressors to male and female intruders. Female intruder was presented in the first day and male intruder was presented in the second day, and vice versa. The order was counter balanced between animals. The latency to first attack and the duration and frequency of attack bouts were observed from the video.

SUPPLEMENTAL REFERENCE

Golden, S.A., Heshmati, M., Flanigan, M., Christoffel, D.J., Guise, K., Pfau, M.L., Aleyasin, H., Menard, C., Zhang, H., Hodes, G.E., Bregman, D., Khibnik, L., Tai, J., Rebusi, N., Krawitz, B., Chaudhury, D., Walsh, J.J., Han, M.H., Shapiro, M.L., Russo, S.J. Basal forebrain projections to the lateral habenula modulate aggression reward. *Nature*. **534**:688-692 (2016).

SUPPLEMENTAL VIDEOS

Supplemental Video 1. Aggressive behavior of CD-1 aggressor male after CNO injection in the conventional RSDS paradigm.

Supplemental Video 2. Aggressive behavior of ERα-Cre aggressor male mice after CNO injection in the adapted RSDS paradigm without social contact.
